# Dynamic relative regional lung strain estimated by computed tomography and electrical impedance tomography in ARDS patients

**DOI:** 10.1186/s13054-023-04748-4

**Published:** 2023-11-24

**Authors:** Roberto Brito, Caio C. A. Morais, Marioli T. Lazo, Dannette V. Guiñez, Abraham I. J. Gajardo, Daniel H. Arellano, Marcelo B. P. Amato, Rodrigo A. Cornejo

**Affiliations:** 1 Unidad de Pacientes Críticos, Departamento de Medicina, Hospital Clínico Universidad de Chile, Dr. Carlos Lorca Tobar 999, Independencia, Santiago Chile; 2 Divisao de Pneumologia, Faculdade de Medicina, Instituto do Coração, Hospital das Clinicas HCFMUSP, Universidade de São Paulo, São Paulo, Brazil; 3 Departamento de Fisioterapia, Universidade Federal de Pernambuco, Recife, Brazil; 4 Programa de Fisiopatología, Facultad de Medicina, Instituto de Ciencias Biomédicas, Universidad de Chile, Santiago, Chile; 5 Departamento de Kinesiología, Facultad de Medicina, Universidad de Chile, Santiago, Chile; 6 Center of Acute Respiratory Critical Illness (ARCI), Santiago, Chile

**Keywords:** Acute respiratory distress syndrome, Strain, Computed tomography, Electrical impedance tomography

## Abstract

**Background:**

In the acute distress respiratory syndrome (ARDS), specific lung regions can be exposed to excessive strain due to heterogeneous disease, gravity-dependent lung collapse and injurious mechanical ventilation. Computed tomography (CT) is the gold standard for regional strain assessment. An alternative tool could be the electrical impedance tomography (EIT). We aimed to determine whether EIT-based methods can predict the dynamic relative regional strain (DRRS) between two levels of end-expiratory pressure (PEEP) in gravity-non-dependent and dependent lung regions.

**Methods:**

Fourteen ARDS patients underwent CT and EIT acquisitions (at end-inspiratory and end-expiratory) at two levels of PEEP: a low-PEEP based on ARDS-net strategy and a high-PEEP titrated according to EIT. Three EIT-based methods for DRRS were compared to relative CT-based strain: (1) the change of the ratio between EIT ventilation and end-expiratory lung impedance in arbitrary units ([Δ*Z*_AU low-PEEP_/EELI_AU low-PEEP_]/[Δ*Z*_AU high-PEEP_/EELI_AU high-PEEP_]), (2) the change of Δ*Z*/EELI ratio calibrated to mL ([Δ*Z*_ml low-PEEP_/EELI_ml low-PEEP_]/[Δ*Z*_ml high-PEEP_/EELI_ml high-PEEP_]) using CT data, and (3) the relative change of ∆*Z*_AU_ (∆*Z*_AU low-PEEP_/∆*Z*_AU high-PEEP_). We performed linear regressions analysis and calculated bias and limits of agreement to assess the performance of DRRS by EIT in comparison with CT.

**Results:**

The DRRS assessed by (Δ*Z*_ml low-PEEP_/EELI_ml low-PEEP_)/(Δ*Z*_ml high-PEEP_/EELI_ml high-PEEP_) and ∆*Z*_AU low-PEEP_/∆*Z*_AU high-PEEP_ showed good relationship and agreement with the CT method (*R*^2^ of 0.9050 and 0.8679, respectively, in non-dependent region; *R*^2^ of 0.8373 and 0.6588, respectively, in dependent region; biases ranging from − 0.11 to 0.51 and limits of agreement ranging from − 0.73 to 1.16 for both methods and lung regions). Conversely, DRRS based on EELI_AU_ ([Δ*Z*_AU low-PEEP_/EELI_AU low-PEEP_]/[Δ*Z*_AU high-PEEP_/EELI_AU high-PEEP_]) exhibited a weak negative relationship and poor agreement with the CT method for both non-dependent and dependent regions (*R*^2^ ~ 0.3; bias of 3.11 and 2.08, and limits of agreement of − 2.13 to 8.34 and from − 1.49 to 5.64, respectively).

**Conclusion:**

Changes in DRRS during a PEEP trial in ARDS patients could be monitored using EIT, based on changes in Δ*Z*_mL_/EELI_ml_ and ∆*Z*_AU_. The relative change ∆*Z*_AU_ offers the advantage of not requiring CT data for calibration.

## Introduction

Dynamic lung strain refers to the deformation of the pulmonary parenchyma during tidal ventilation (V_T_) relative to the end-expiratory lung volume (EELV) [1, 2]. Specific lung regions can be exposed to excessive strain due to heterogeneous disease, gravity-dependent lung collapse, and injurious mechanical ventilation. This excessive regional strain correlates with worsening local inflammation in acute respiratory distress syndrome (ARDS) [2–4].

The gold-standard method to assess regional strain is computed tomography (CT). However, this is a time-consuming procedure that exposes the patient to X-ray radiation [5]. As an alternative, electrical impedance tomography (EIT), a bedside radiation-free method, has been proposed for assessing regional strain [6]. In a proof-of-concept study, our group demonstrated a strong association between changes in strain measured by CT and changes in electrical impedance (Δ*Z*) at two levels of positive end-expiratory pressure (PEEP) [7].

A novel method of EIT-based strain was proposed by Gogniat et al. [6] calculating the relative change in lung strain at two levels of PEEP by dividing Δ*Z* by end-expiratory lung impedance (EELI), a surrogate of EELV. This approach is compelling as it shares similarities with the classical CT-based strain method. However, caution is warranted in utilizing EELI in arbitrary units (A.U., the standard EIT unit), which may provide inaccurate measurements of strain due to “arbitrariness” of the absolute value at the end of expiration, such as the possibility of values close to zero or even negative (producing a non-physiological strain). In addition, a validation study comparing the change of regional strain measured by CT and EIT is lacking.

Therefore, we aimed to determine whether EIT-based methods, including the recent ΔZ/EELI approach in A.U., can predict changes in dynamic regional strain between two levels of PEEP measured by CT in gravity-non-dependent and dependent lung regions in ARDS patients.

## Methods

This study involved mechanically ventilated patients with ARDS under deep sedation and neuromuscular blockade on volume-controlled ventilation (VCV) with V_T_ of 6 ml/kg of predicted body weight. The study was approved by the Ethics Committee of Hospital Clínico Universidad de Chile (N.027/2016). Dynamic strain was assessed in gravity-non-dependent and dependent lung regions using whole-lung low radiation CT [7] and EIT (Enlight 1800, Timpel Medical, Brazil) simultaneously. These regions-of-interest were selected due to physiological relevance [8] and simple clinical applicability. Part of the CT and EIT data used in this study were obtained from a previous investigation [7]. Data collection was performed through end-expiration and end-inspiration breath-holds at two PEEP levels, applied in a random order according to the ARDS-net strategy (low-PEEP) [9] and to the EIT (high-PEEP). The latter was defined as the PEEP associated with the lowest combination of collapse and overdistension during a decremental PEEP trial after a recruitment maneuver [7, 10]. The end-inspiration holds were performed by configuring a continuous positive airway pressure (CPAP) level similar to the plateau pressure, while end-expiratory holds utilized CPAP at the same PEEP total level. The reference frames for the EIT image reconstruction were based on the PEEP level defined by the ICU team before the performed PEEP titration.

The CT strain (Strain_CT_) was calculated as the ratio between V_T_ and EELV. The relative change in Strain_CT_ between low-PEEP and high-PEEP, termed dynamic relative regional strain (DRRS), was defined as (Strain_CT low-PEEP_/Strain_CT high-PEEP_) for each region-of-interest. The EIT-based strain was assessed according to the following methods:

1. The relative change of ΔZ/EELI ratio in A.U. between the lowest and the highest value of PEEP ([ΔZ_AU low-PEEP_/EELI_AU low-PEEP_]/[ΔZ_AU high-PEEP_/EELI_AU high-PEEP_]).
2. The relative change of ΔZ/EELI ratio in mL between the lowest and the highest value of PEEP ([ΔZ_ml low-PEEP_/EELI_ml low-PEEP_]/[ΔZ_ml high-PEEP_/EELI_ml high-PEEP_]). For this calculation, we converted the regional EELI in mililiters (EELI_ml_) at low-PEEP using corresponding EELV measured by CT. The ΔZ_ml_ was computed multiplying the ΔZ_AU_ by the ratio of V_T_/ΔZ_AU_ at low-PEEP. Finally, the EELI_ml_ at high-PEEP was estimated from the sum of EELI_mL_ at low-PEEP and ΔEELI_ml_ calculated multiplying the ΔEELI_AU_ by the ratio of V_T_/ΔZ_AU_ at low-PEEP [11].
3. the relative change of ∆Z_AU_ between the lowest and the highest value of PEEP (∆Z_AU low-PEEP_ /∆Z_AU high-PEEP_) [7].

**Fig. 1:**
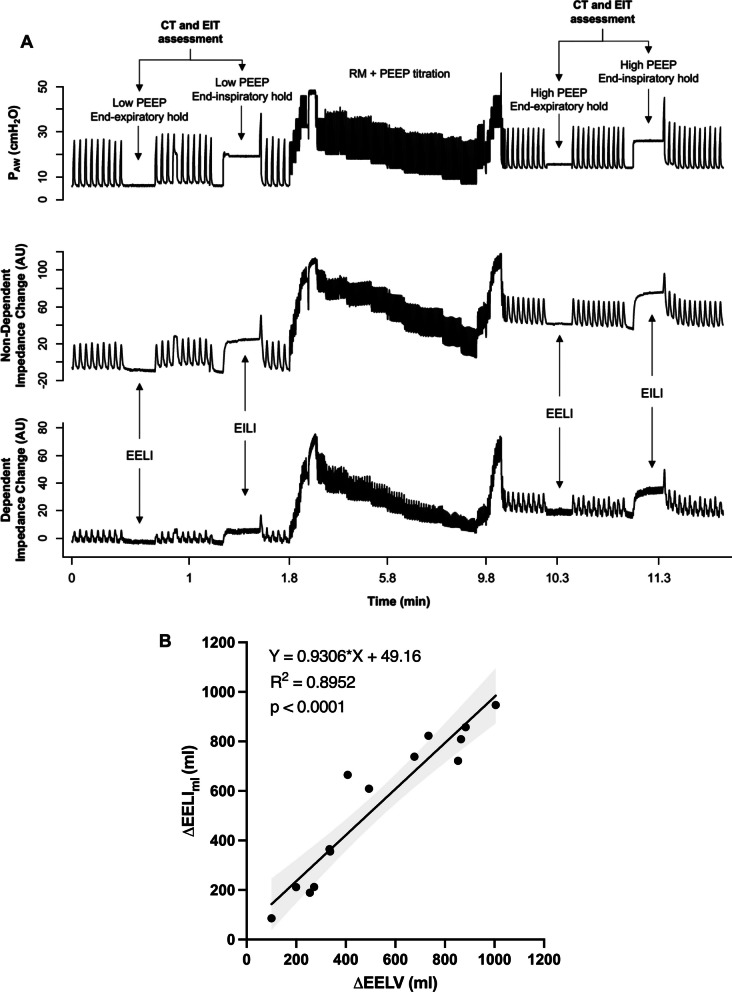
Experimental protocol and correlation between changes in lung volume by computed tomography (CT) and electrical impedance tomography (EIT) related data. **A** Experimental protocol of a representative case. Traces of airway pressure (*upper row*), impedance change in non-dependent (*middle row*) and dependent (*lower row*) lung regions during the study timeframe are shown. Two levels of PEEP were applied in random order (in this representative case, first low-PEEP and then high-PEEP, after a recruitment maneuver). At both PEEP levels, end-expiratory and end-inspiratory holds were applied to obtain positive end-expiratory pressure and end-expiratory lung impedance (EELI), and airway plateau pressure and end-inspiratory lung impedance (EILI), respectively. The impedance change (Δ*Z*) corresponded to the difference between EILI and EELI. CT and EIT assessments were performed at the same time. **B** Linear correlation between delta end-expiratory lung volume (ΔEELV) CT-measured and the change of lung volume obtained from changes in end-expiratory lung impedance (ΔEELI_ml_)

The summary of the protocol is shown in Fig. 1A. We performed linear regressions analysis and calculated bias and limits of agreement to assess the performance of DRRS by EIT in comparison with CT.

## Results

Fourteen patients (age 67 [60–76] years, 8 males) were included. Their worst PaO_2_:FiO_2_ ratio during the acute phase of ARDS was 129 [96–167] mmHg. At the study entry, the mechanical ventilation time was 8 [4–12] days and PaO_2_:FiO_2_ ratio was 235 [210–274] mmHg with FiO_2_ 0.35 [0.30–0.36]. The median low-PEEP and high-PEEP values were 6 [5–7] cm H_2_O and 12 [10–14] cm H_2_O, respectively.

Global EELV was 1300 [1064–1706] ml at low-PEEP and 1901 [1472–2463] ml at high-PEEP. Global EELI was − 14.67 [− 28.1 to − 11.86] A.U. at low-PEEP and 10.91 [− 7.57 to 32.53] A.U. at high-PEEP. The ΔEELI_ml_, induced by PEEP changes, demonstrated a strong association with the ΔEELV detected by CT (Fig. 1B).

**Fig. 2:**
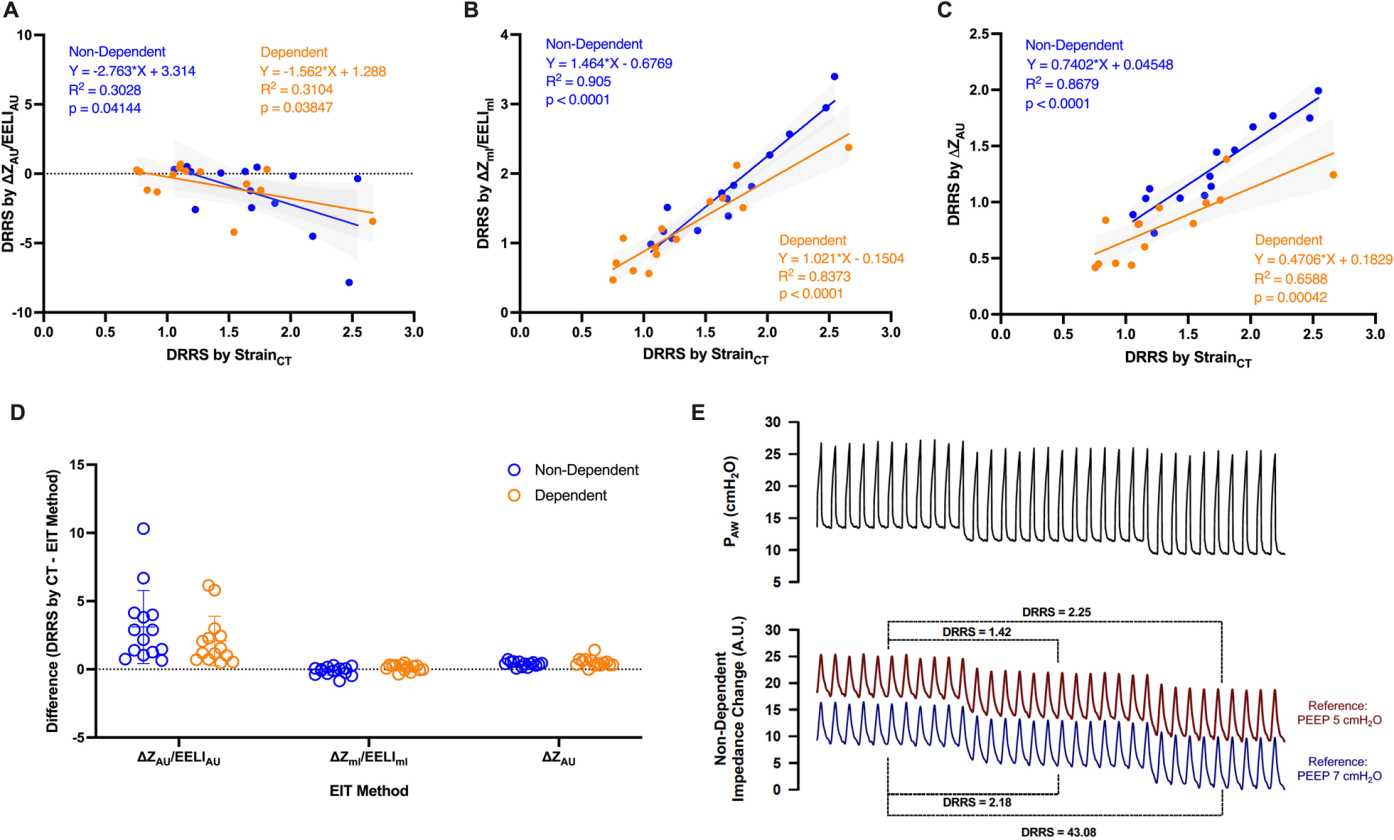
Association and agreement between CT-based DRRS and the different EIT-based methods evaluated in non-dependent and dependent lung regions. **A** Association between DRRS by CT and DRRS by Δ*Z*_AU_/EELI_AU_. **B** Association between DRRS by CT and DRRS by Δ*Z*_ml_/EELI_ml_. **C** Association between DRRS by CT and DRRS by Δ*Z*_AU_ (For **A–C** individual values and linear regression curves with 95% confidence bands for both regions analyzed are shown). **D** Agreement between CT-based DRRS and the different EIT-based methods. For this analysis, the differences between the individual value of CT-based DRRS and the respective EIT-based DRRS values are shown with mean and standard deviation for each EIT method. **E** Illustration of changes in EELI induced by PEEP using different references for EIT reconstruction (PEEP 5 cmH_2_O and PEEP 7 cmH_2_O). Note that modifying the reference results in arbitrary DRRS values (both in absolute levels and the differences between the PEEP steps), with an increase in reference EELI values which leads to lower DRRS

We observed a negative association between DRRS by ∆Z_AU_/EELI_AU_ and DRRS by Strain_CT_ in both lung regions (Fig. 2A; *R*^2^ ~ 0.3), and a poor agreement for both non-dependent and dependent regions (bias of 3.11 and 2.08, and limits of agreement of − 2.13 to 8.34 and from − 1.49 to 5.64, respectively).

On the other hand, DRRS based on ∆Z_ml_/EELI_ml_ and ∆Z_AU_ showed good relationship and agreement with the reference method in both lung regions, with biases ranging from − 0.11 to 0.51 and limits of agreement ranging from − 0.73 to 1.16 (Fig. 2C–E).

## Discussion

This study demonstrated that DRRS estimation between two levels of PEEP is feasible at the bedside using EIT. The DRRS based on changes in ∆*Z*_ml_/EELI_ml_ accurately predicts the change in lung strain assessed by CT in different gravitational lung regions. Our data also suggests that the relative change in ∆*Z*_AU_ induced by PEEP changes (∆*Z*_AU low-PEEP_/∆*Z*_AU high-PEEP_) can be used as a surrogate of DRRS.

However, the DRRS based on changes in Δ*Z*_AU_/EELI_AU_ exhibited a behavior that contradicts biophysically principles when EELI_AU_ is negative. The negative value of EELI_AU_ is a frequent finding; indeed, its absolute value depends on the clinical condition at the start of EIT recordings (i.e., the reference frame), and it varies significantly among subjects, and even within the same subject [12, 13]. EELI_AU_ is also influenced by changes in intrathoracic blood volume or fluid status [14]. From a physical perspective, the absolute values of EELI are intrinsically meaningless. Its value should be exclusively derived from its linear relationship with changes in lung air content. Furthermore, any attempt to avoid negative EELI_AU_ values, such as using a lower level of PEEP as a reference value for EIT reconstruction, will not yield meaningful DRRS value. The higher the adjustment in EELI_AU_, the lower the DRRS value (see Fig. 2E). Therefore, relying on EELI measurements in arbitrary units is impractical for quantifying strain independently of the EIT reference.

The outperformance of the relative changes in ∆*Z*_ml_/EELI_ml_ for assessing DRRS was expected because this approach intrinsically cancelled the influence of absolute values of EELI (A.U.), retaining only its relative changes to a reference condition. A strong correlation between ∆EELV and ∆EELI during a PEEP trial is a fundamental requirement for employing this approach [11, 15]. To achieve this, we avoided changes in patient positioning between PEEP steps and also any bolus of intravenous infusions. The current study used for first time CT data to perform the correlations between ∆EELV and ∆EELI_ml_ at global and regional level. Our findings reinforce the close association between CT and EIT for EELV-related data, validating its use for calculating DRRS. The major limitation of this approach is the requirement of a baseline CT data for calibration.

An alternative is using the relative changes in ∆*Z*_AU_ as a surrogate of DRRS. The ability of ∆Z to capture the change of strain in response to increase in PEEP was also suggested in the Gogniat et al. [6] study. Despite similar V_T_ at PEEP 15 and ZEEP in the pigs (333 ± 71 ml and 334 ± 74, respectively, *p* = non-significant), ∆*Z*_AU_ significantly increased between PEEP 15 and ZEEP (from 0.35 ± 0.90 to 0.46 ± 0.14 A.U., *p* < 0.05). Therefore, ∆*Z*_AU_ in lung parenchyma exhibits a behavior similar to that observed in other biological tissues in response to mechanical deformation [16]. However, it is essential to acknowledge two important points: (1) regional ΔZ_AU_ may be influenced by the regional redistribution of ventilation induced by higher PEEP levels, and (2) the ΔZ_AU_ ratio at low-PEEP/high-PEEP is not a direct measurement of strain, as traditionally established by the ratio V_T_/EELV.

Our findings must be interpreted with caution due to some limitations: (1) being a clinical study of limited size; (2) only two levels of PEEP were evaluated and (3) the selection of regions-of-interest was based on the gravity gradient, not accounting for individualized lung injury distribution.

In conclusion, changes in DRRS during a PEEP trial in ARDS patients could be monitored using EIT, based on changes in Δ*Z*_mL_/EELI_ml_ and ∆*Z*_AU_. While the ∆*Z* method may be slightly less precise compared to the standard Δ*Z*_mL_/EELI_ml_ method, it offers the advantage of not requiring any CT data for calibration. Further research is needed to explore the clinical significance of the Δ*Z*_AU_ _low-PEEP_/Δ*Z*_AU_ _high-PEEP_ method in lung protection, as well as its comparison with other VILI determinants like transpulmonary pressure.

## Data Availability

The datasets and materials used and/or analyzed during the current study are available from the corresponding author on reasonable request.

## Abbreviations

ARDS: Acute respiratory distress syndrome
V_T_: Tidal ventilation
EELV: End-expiratory lung volume
CT: Computed tomography
EIT: Electrical impedance tomography
Δ*Z*: Tidal change in electrical impedance
PEEP: Positive end-expiratory pressure
EELI: End-expiratory lung impedance
EILI: End-inspiratory lung impedance
A.U.: Arbitrary units
VCV: Volume-controlled ventilation
DRRS: Dynamic relative regional strain
SOFA score: Sequential organ failure assessment score
PaO_2_FiO_2_ ratio: Ratio of arterial partial pressure of oxygen to inspired oxygen fraction
